# Same-side recurrence of unilateral multiple evanescent white dot syndrome following the thermal laser photocoagulation for inflammatory macular neovascularization

**DOI:** 10.3205/oc000250

**Published:** 2025-05-02

**Authors:** Barbaros Hayrettin Ünlü, Omer Karti, Ayse Bozkurt Oflaz, Erk Atlay, Ali Osman Saatci

**Affiliations:** 1 Dokuz Eylul University, Department of Ophthalmology, İzmir, Turkey; 2 Selcuk University, Department of Ophthalmology, Konya, Turkey

**Keywords:** choriodal neovascularization, fundus autofluorescence, laser photocoagulation, macular neovascularization, multifocal ERG, multiple evanescent white dot syndrome

## Abstract

**Purpose:**

We report the same side recurrence of multiple evanescent white dot syndrome (MEWDS) subsequent to 532 nm laser treatment for the macular neovascularization (MNV) associated with the first MEWDS episode.

**Method:**

Retrospective case documentation with the multimodal imaging.

**Result:**

A 24-year-old otherwise healthy woman who was diagnosed as having left MEWDS four years ago was re-examined for a visual disturbance of the duration of one month in the same eye. Fundus evaluation led us to the diagnosis of left extrafoveal inflammatory MNV. Surprisingly, she developed further visual deterioration a month following the uneventful 532 nm laser photocoagulation in her left eye. Fundus examination and multimodal imaging tests confirmed the recurrent MEWDS after full negative laboratory work-up. Visual acuity and fundus changes were improved with the help of a short course oral steroid therapy.

**Conclusion:**

MEWDS can very rarely recur and thermal laser photocoagulation may be a possible triggering factor.

## Introduction

Multiple evanescent white dot syndrome (MEWDS) is a rare, primarily unilateral inflammatory disease that was initially described by Jampol et al. in 1984 [1], [2]. This disorder typically affects young to middle-aged females. The characteristic lesions consist of multifocal, small white dots located at the level of the outer retina or retinal pigment epithelium (RPE), usually found in the posterior pole or mid-peripheral retina [2], [3].

MEWDS typically presents with a flu-like prodrome. Possible triggers include viral infections, vaccinations, autoimmune mechanisms, and genetic susceptibility. However, the exact cause and mechanism of this condition is still not fully understood [3], [4]. There is an ongoing debate about the main target of inflammation, with some arguing that it mostly affects the photoreceptors, while others classify it as a primary inflammatory choriocapillaropathy [3], [4].

Although initially characterised as a unilateral disorder with a self-limiting course, MEWDS can manifest bilaterally, may recur and is even complicated with inflammatory type macular neovascularization (MNV) [3], [5]. In this report, we present a unilateral MEWDS case thatrecurred following the thermal laser photocoagulation therapy for the MNV formation four years after the initial episode. To our best knowledge, this is the first reported case of presumed laser photocoagulation related recurrence of MEWDS.

## Case description

**Figure 1:**
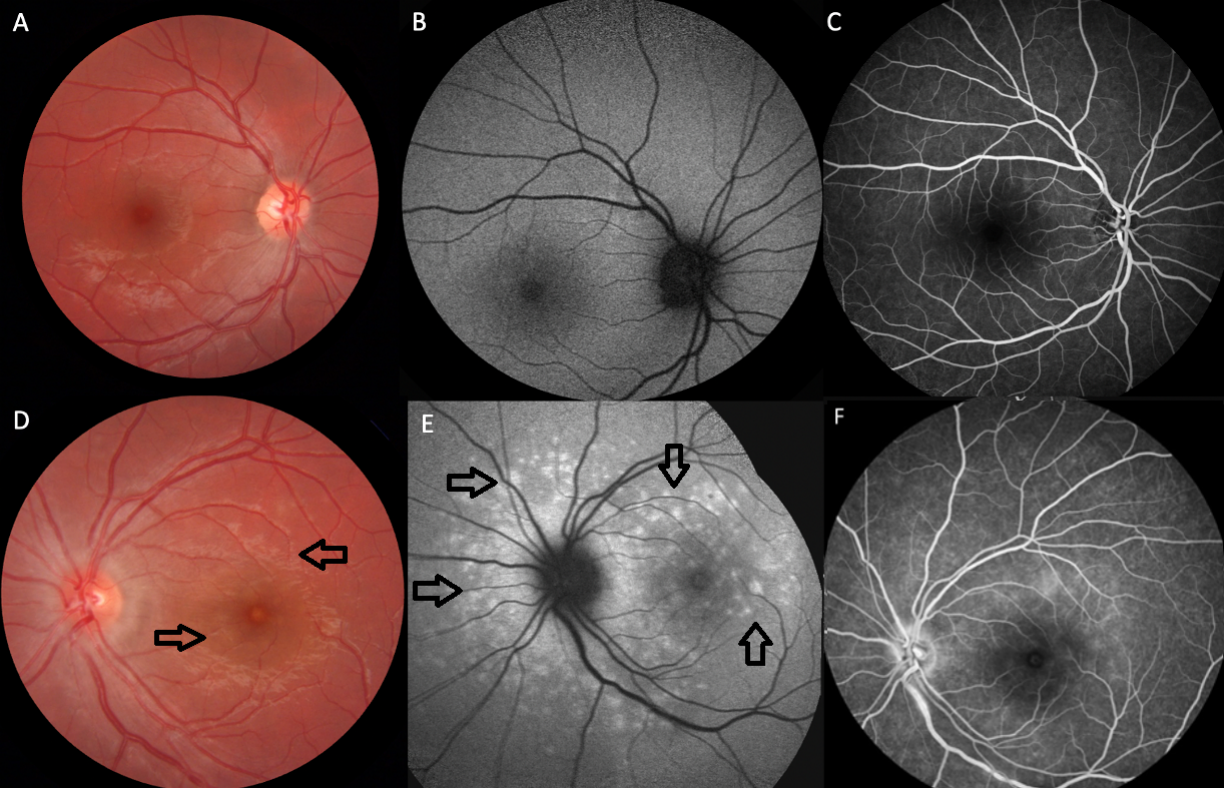
Color fundus (CF), fundus autofluorescence (FAF), and fluorescein angiography (FA) images of the patient at the time of initial MEWDS diagnosis in 2019. CF photo of the right eye (A) displayed no abnormality, while there were small whitish dots scattered around the optic disc and throughout the posterior pole (black arrows) in the left eye (D). Right FAF image (B) exhibited the normal appearance, whereas hyper-autofluorescent areas corresponding to the fundus alterations (black arrows) were observed in the left eye (E). FA displayed no changes in the right eye (C), whereas left eye showed hyperfluorescent lesions corresponding to the FAF alterations (F).

A 24-year-old otherwise healthy woman was examined due to blurred vision and metamorphopsia in her left eye in January 2024. Notably, she received a diagnosis of left MEWDS at our department in April 2019. At that time, her best-corrected Snellen visual acuity (BCVA) was 10/10 in both eyes. Slit-lamp examination was unremarkable bilaterally. While right fundus was normal, there were multiple small whitish dots scattered around the optic disc and throughout the posterior pole in the left eye (Figure 1A and D (Fig. 1)). While the fundus autofluorescence (FAF) image was normal in the right eye, there were circumpapillary hyperautofluorescent areas at the left posterior pole (Figure 1B and E (Fig. 1)). Fluorescein angiogram (FA) of the right fundus was normal whereas there were scattered subtle hyperfluorescent lesions corresponding to the fundus autofluorescence alterations in both early and late phases of the left angiogram (Figure 1C and F (Fig. 1)). We did not recommend any treatment as our diagnosis was unilateral MEWDS.

**Figure 2:**
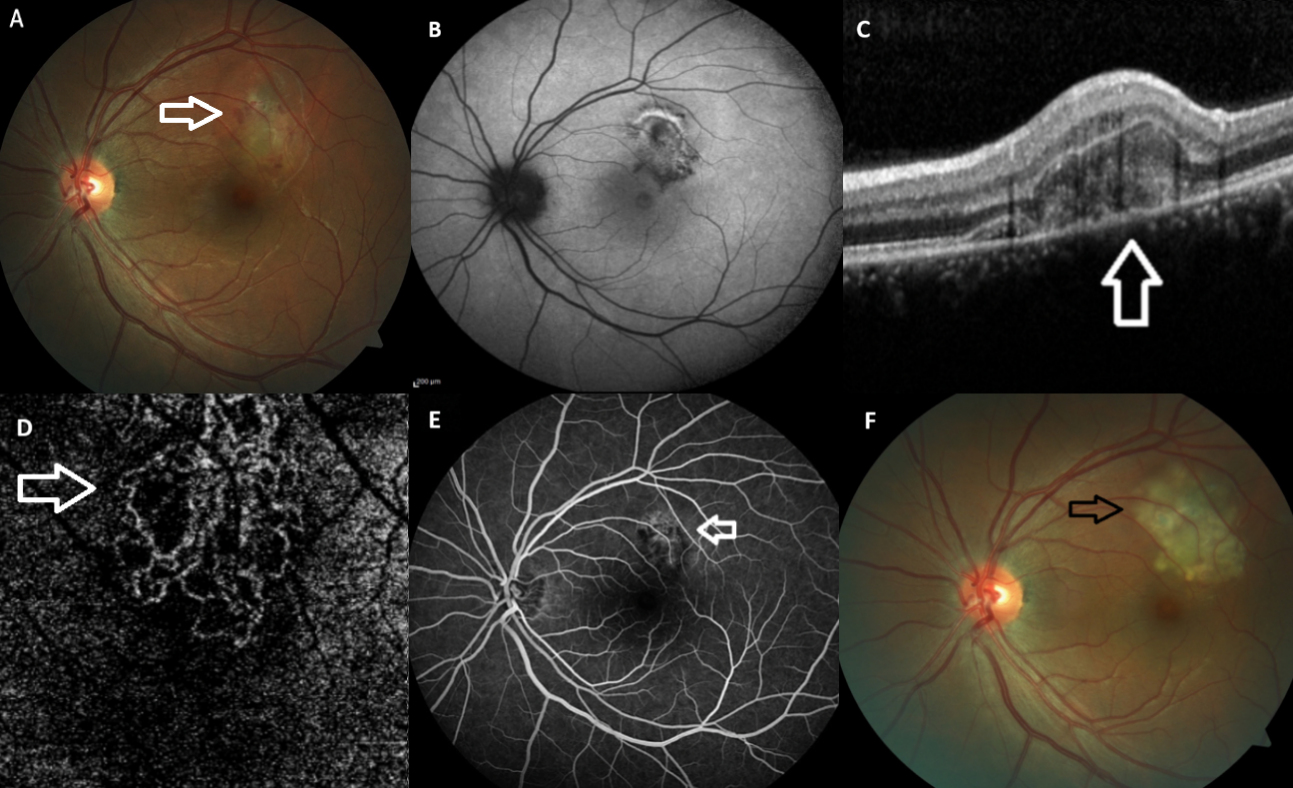
Left eye, January 2024. Color fundus (CF) photo (A) displaying the subretinal hemorrhage and macular edema (white arrow) at the superior perifoveal area. Fundus autofluorescence image (B) revealing the hypo/hyper-autofluorescent areas corresponding to the fundus alterations. Macular neovascularization (MNV) with subretinal fluid and subfoveal hyperreflective material (white arrow) was detected on optical coherence tomographic (OCT) section passing through the lesion (C). OCT angiography exhibiting the abnormal vascular network (white arrow) at the outer retinal layer consistent with a type 2 MNV (D). Fluorescein angiography (E) demonstrating the hyperfluorescence related to MNV with dye leakage (white arrow). CF image (F) showing the thermal laser photocoagulation treatment (black arrow) over the MNV area.

**Figure 3:**
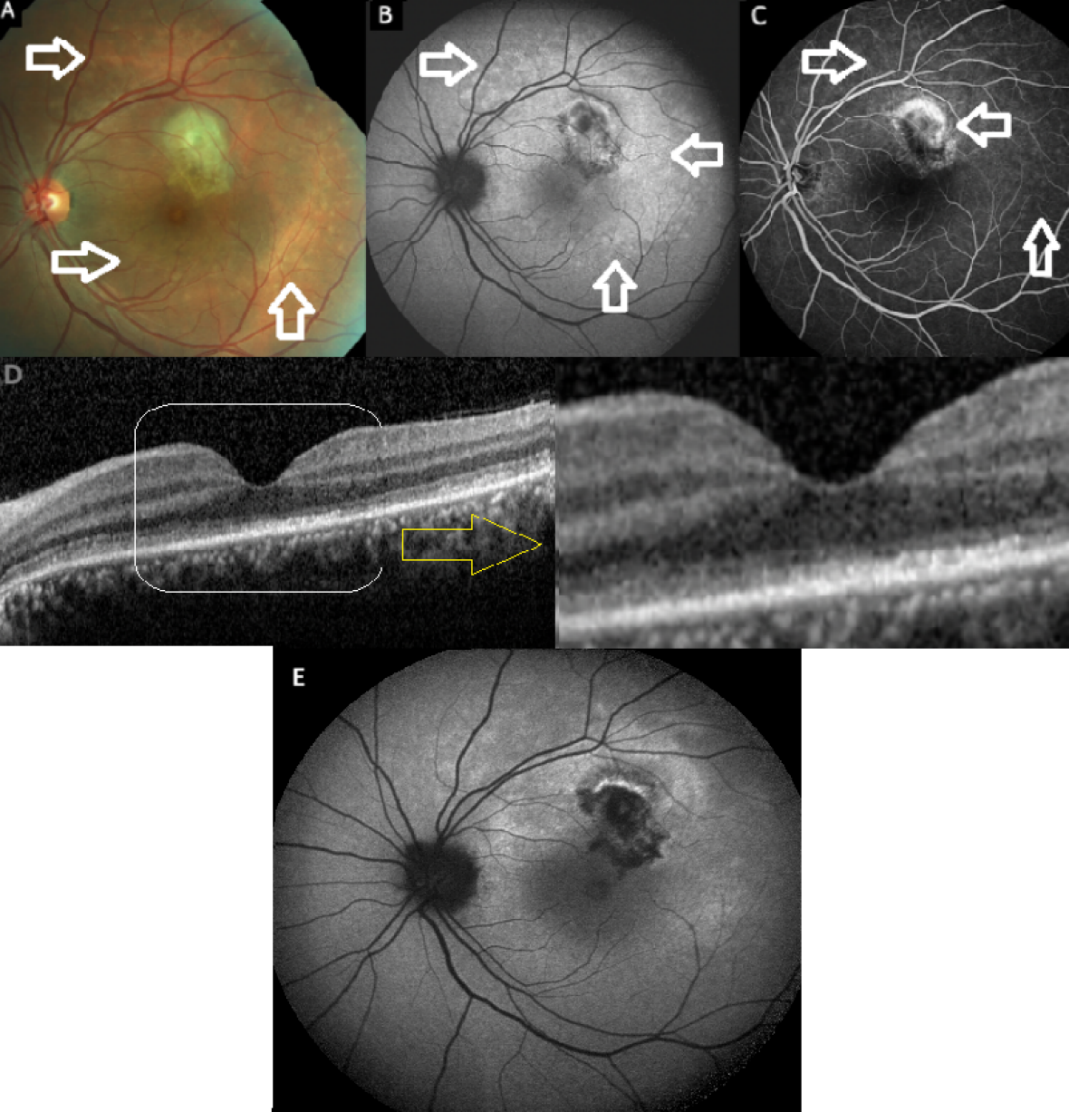
Left eye. Color fundus (CF), fundus autofluorescence (FAF), optical coherence tomographic (OCT), and fluorescein angiography (FA) images at the time of MEWDS recurrence. CF image (A) revealing the multiple white spots at the posterior pole (white arrows). FAF image (B) exhibiting some hyper-autofluorescent areas (white arrows) corresponding to the fundus alterations. FA delineating the hyperfluorescent lesions (white arrows) corresponding to the FAF alterations (C). OCT demonstrating the disruption of the ellipsoid zone and retinal pigment epithelium with subfoveal hyperreflective materials (D). FAF picture (E) indicating a prominent resolution of hyperautofluorescent lesions after two months of the treatment.

The patient was reexamined with a new-onset visual disturbance in her left eye of the duration of one month in January 2024. Her BCVA was 10/10 in both eyes. Slit-lamp examination was unremarkable and intraocular pressure was within normal limits bilaterally. While right fundus appeared normal, there was a greyish looking area with some subretinal hemorrhage at the upper macula (Figure 2A (Fig. 2)). The FAF image revealed no sign of hyper-autofluorescent changes related to the patient’s first MEWDS episode at the posterior pole (Figure 2B (Fig. 2)), but optical coherence tomography (OCT) revealed the presence of MNV together with subretinal fluid and hyperreflective material (Figure 2C (Fig. 2)). En face OCT angiography showed an abnormal vascular network at the outer retinal layer consistent with type 2 MNV (Figure 2D (Fig. 2)). FA revealed hyperfluorescence of MNV characterized with the leakage (Figure 2E (Fig. 2)). The patient was diagnosed with MNV secondary to MEWDS and received 532 nm laser photocoagulation treatment on the same day (Figure 2F (Fig. 2)). A month later, in February 2024, the patient’s left visual acuity dropped to counting fingers at one meter. The anterior segment examination was still unremarkable in both eyes. Fundoscopy revealed multiple very subtle whitish spots at the left posterior pole (Figure 3A (Fig. 3)). FAF images demonstrated circumpapillary hyperautofluorescent areas at the left fundus (Figure 3B (Fig. 3)). FA demonstrated hyperfluorescent staining of the same areas with inactive looking MNV area (Figure 3C (Fig. 3)). The OCT revealed diffuse disruption at the ellipsoid zone and punctate hyperreflective lesions of varying sizes in the outer retina, indicative of a recurrence of MEWDS (Figure 3D (Fig. 3)). Infectious etiology was ruled out including syphilis serology and Quantiferon test. As the vision was poor, a short course of oral methylprednisolone was commenced (60 mg) with a rapid taper. Two months later, BCVA was 10/10 in the right eye and improved to 7/10 in the left eye. The FAF image revealed the marked resolution of the hyperautofluorescent lesions (Figure 3E (Fig. 3)).

Macular integrity was tested using a scanning laser ophthalmoscope (SLO) microperimeter (MAIA; CenterVue-iCare, Padova, Italy) at the time of MEWDS recurrence. There was a laser related scotoma with a 4-2 strategy mode in the upper temporal region of the left macula. The average threshold value was 12.6 decibels (dB). The patient’s fixation points were found to be distributed (P1=22%, P2=61%, 63% bivariate contour ellipse area (BCEA)=14.5°², 95% BCEA=43.5°²). Two months later, microperimetry was repeated, and the scotoma was shrunken on the colored scale used to assess the patient’s sensitivity and the average threshold value was increased to 21.7 dB. Among the tests, fixation behaviors produced the most consistent results (P1=98%, P2=100%, 63% BCEA=0.4°², 95% BCEA=1.2°²).

## Discussion

This case report clearly documents for the first time the recurrence of MEWDS in a patient who underwent thermal laser photocoagulation for the treatment of MNV that occurred as the complication of the first MEWDS episode. MEWDS is currently classified as “typical/primary” form and “atypical/secondary” form [6]. The presentation of primary MEWDS entails a singular acute process, characterized by spontaneous resolution without enduring structural anatomical disruption at the outer posterior fundus or compromise of the visual function [6]. The recurrence of primary MEWDS is rarely reported [3], [5], [7]. Ramakrishnan et al. [5] noted a recurrence only in 10 of 73 MEWDS patients (14%) in a comprehensive retrospective cohort study. However, the study did not sort out the potential factors associated with MEWDS recurrence [5].

On the other hand, secondary MEWDS often presents in association with the other posterior segment disorders such as macular diseases or iatrogenic retinal injuries. Multifocal choroiditis (MFC) is the most frequently observed entity among the triggering disorder for the secondary MEWDS besides the entities Best’s vitelliform dystrophy, acute zonal occult outer retinopathy, toxoplasmosis chorioretinitis, pseudoxanthoma elasticum with angioid streaks, retinal trauma, retinopexy, retinal detachment, subretinal hemorrhage, cyberknife stereotactic radiotherapy for choroidal malignant melanoma, choroidal granuloma, and MNV [6], [8], [9], [10]. Cicinelli et al. [10] also described patients with MEWDS-like reactions (secondary MEWDS) concurrent with various posterior segment abnormalities such as Best vitelliform dystrophy, angioid streaks, retinal detachment surgery, undetermined retinochoroiditis, suggesting a potential link between the disruption of macular RPE or Bruch’s membrane and acute inflammatory events. In our case, the recurrence of MEWDS occurred following the thermal laser photocoagulation. This phenomenon has not been previously described in the literature. The clinical sequence in our case insinuated that thermal laser administration causing RPE and Bruch’s membrane disruption might have paved the path for MEWDS recurrence.

The “uveitis-related CNV” or “inflammatory CNV (iCNV)” represents a rare complication of primary MEWDS, typically observed in cases of MFC or punctate inner choroiditis presenting with MEWDS-like reactions [11], [12], [13]. Despite the precise etiology of iCNV remaining elusive, dysregulation between stimulating and inhibitory soluble mediators produced by the RPE appears to be a primary instigating factor in its development. The interplay of cytokines, in conjunction with vascular endothelial growth factor (VEGF), leads to compromised permeability and aberrant angiogenesis [11].

The optimal management of iCNV remains uncertain due to its complexity and lack of a straightforward algorithm. Effective management of active inflammation with steroids and/or immunosuppressive agents or biologics is crucial for achieving favorable outcomes [11]. VEGF inhibitors have become the primary adjunctive treatment due to their superior anatomical and functional outcomes and low recurrence rate of iCNV [11], [13]. In our case, MEWDS-related MNV was treated with thermal laser photocoagulation due to its extrafoveal location. However, after experiencing the MEWDS recurrence in the present case it can be speculated that anti-VEGF therapy might be preferred over thermal laser photocoagulation even if the MNV lesion is extrafoveal in order to avoid its possible triggering effect.

## Conclusion

Primary MEWDS may rarely recur, and the laser photocoagulation for the MEWDS related MNV could potentially trigger a MEWDS recurrence. Thus, anti-VEGF therapy is better preferred over laser photocoagulation even for extrafoveal MNVs in this group of patients to avoid the triggering effect.

## Notes

### Author’s ORCID

Ali Osman Saatci: 0000-0001-6848-7239

### Patient consent

Patient consent was obtained for publication. In addition, this report does not contain any personal information that could lead to the identification of the patient.

### Competing interests

The authors declare that they have no competing interests.
